# Alcohol and the Risk of Railway Suicide

**DOI:** 10.3390/ijerph17197003

**Published:** 2020-09-24

**Authors:** Dorota Lasota, Ahmed Al-Wathinani, Paweł Krajewski, Dagmara Mirowska-Guzel, Krzysztof Goniewicz, Attila J. Hertelendy, Riyadh A. Alhazmi, Witold Pawłowski, Amir Khorram-Manesh, Mariusz Goniewicz

**Affiliations:** 1 Department of Experimental and Clinical Pharmacology, Medical University of Warsaw, Banacha 1b Street, 02097 Warsaw, Poland; dlasota@wum.edu.pl; 2 Department of Emergency Medical Services, Prince Sultan Bin Abdulaziz College for Emergency Medical Services, King Saud University, Riyadh 11451, Saudi Arabia; ahmalotaibi@ksu.edu.sa (A.A.-W.); rialhazmi@ksu.edu.sa (R.A.A.); 3 Department of Forensic Medicine, Medical University of Warsaw, 50368 Warsaw, Poland; pkrajewski@wum.edu.pl; 4 Department of Aviation Security, Military University of Aviation, 08521 Dęblin, Poland; k.goniewicz@law.mil.pl; 5 Department of Information Systems and Business Analytics, College of Business, Florida International University, Miami, FL 33174, USA; ahertele@fiu.edu; 6 Department of Emergency Medicine, Medical University of Lublin, 20081 Lublin, Poland; witold.pawlowskl@dr.com (W.P.); mariusz.goniewicz@umlub.pl (M.G.); 7 Institute of Clinical Sciences, Department of Surgery, Sahlgrenska Academy, Gothenburg University, 41345 Gothenburg, Sweden; amir.khorram-manesh@surgery.gu.se; 8 Department of Development and Research, Armed Forces Center for Defense Medicine, Västra Frölunda, 42676 Gothenburg, Sweden

**Keywords:** alcohol, ethanol, suicide, railway suicide, railway accidents

## Abstract

Suicide is one of the ten most common causes of death in the world. Of all deaths from suicide, 22% can be attributed to the use of alcohol, which means that every fifth suicide would not occur if alcohol were not consumed by the population. People under the influence of alcohol choose more radical and effective methods of dying by suicide, e.g., throwing themselves under a moving vehicle, such as a train. The presented analysis aimed to determine important risk factors affecting railway suicide in Poland and their relation to the state of alcohol intoxication of the victims, and the relationship between ethyl alcohol consumption and the phenomenon of suicide. Documentation obtained from the Department of Forensic Medicine at the Medical University of Warsaw, in the form of death registers and forensic medical records concerning examination and autopsy, was analyzed. This made it possible to identify suicide victims from among pedestrian victims of railway accidents recorded during the period under study. The research was carried out using unidimensional and multidimensional statistical analyses with IBM SPSS Statistics, version 25. Sober suicide victims were statistically significantly older than victims under the influence of alcohol; alcohol concentration was correlated with the age of the victims—the older the victims were, the higher the alcohol concentration. A significantly higher number of deaths attributed to suicide by sober victims was observed in autumn compared to other seasons. Multidimensional analysis showed a statistically significant effect of age and season on the probability of dying by suicide under the influence of alcohol—this probability decreases with the age of the victims and is also significantly lower in autumn. The observed relationship between age and the presence of alcohol in suicide victims can be the cause of railway suicides. Knowledge of the mechanisms of seasonal variability of suicidal behavior can help to develop effective strategies to prevent railway suicides. It is necessary to improve the system of reporting railway suicides, as only reliable statistics provide the possibility of assessing both the scale of the problem and the effectiveness of actions taken.

## 1. Introduction

Suicide is one of the ten most common causes of death in the world, and the social and economic consequences of suicides are comparable to those observed concerning diseases. According to the World Health Organization (WHO), over 800,000 people die by suicide annually, representing one person every 40 s [1]. Suicide is the 15th leading cause of death globally, accounting for 1.4% of all deaths. The global suicide rate is 11.4 per 100,000 people (15.0/100,000 for males and 8.0/100,000 for females) [2]. Suicide rates differ between different countries and geographical regions [3,4]. According to WHO data from 2016, Poland, with an annual suicide rate of 16.2/100,000, was ranked 22nd in the world [5].

Of all psychoactive substances, alcohol is the strongest predisposing factor for impulsive and aggressive behavior. Regardless of the nature of alcohol use disorder (AUD), alcohol is an important risk factor for suicidal behavior. Research shows that 18% to 66% of suicide victims are under the influence of alcohol at the time of death, while in 1/5 of them, alcoholic intoxication can be observed [6,7] Ho et al. observed that elderly people who died of suicide and had a past history of suicidal behavior were more likely to suffer from major psychiatric disorders, encounter social problems in life, and have alcohol detected in the blood toxicology report at autopsy [8]. Within the population, the influence of ethyl alcohol on the phenomenon of suicide has been confirmed by a positive correlation between the amount of alcohol consumed and suicide rates [9]. Of all deaths from suicide, 22% can be attributed to the use of alcohol, which means that every fifth suicide would not occur if the population did not consume alcohol [10]. The consumption of alcohol before carrying out suicide presents a higher risk of dying by suicide than alcohol abuse or addiction alone. On the other hand, chronic alcohol consumption leads to dysfunctions leading to suicidal behavior, as well as dysfunctions of the neurotransmission system in the central nervous system, including a decrease in the activity of the serotonergic system, which is characterized by an increased frequency of suicidal behaviors [6,11,12]. In the majority of all suicides, the simultaneous co-occurrence of more than one mental disorder is observed, wherein the highest risk of suicide is associated with alcohol dependence and depressive disorders [13]. It has not been unequivocally established whether depression appears first or vice versa—alcohol dependence precedes the occurrence of full-blown depression [14]. At the same time, people with depression “self-medicate”, using alcohol in attempts to change negative emotions, cope with low mood, and a sense of hopelessness. This results in alcohol dependence (addiction), which is a phenomenon affecting both sexes, but emphasized more in women [15,16]. Suicide on railroad tracks is defined as an attempt to take one’s own life by trespassing on the track or lying on the track in front of a moving rail vehicle (train, locomotive, trolley); an act of deliberately wounding oneself leading to death [17]. To take one’s own life, those who die by suicide use a variety of methods. According to Polish Police Headquarters statistics for 2019, 5255 deaths by suicide were recorded. These were most often due to hanging (80.68%), but also jumping from heights (6.28%) and jumping under a moving vehicle (2.51%), e.g., under an oncoming train [18,19,20]. Entering the path of a moving train is a method that is considered a sure and quick death and is easily accessible and does not threaten the lives of third parties [20,21]. The factors which contribute to this efficacy are the long braking distance of the train, its large mass, inability to change direction when an obstacle is noticed, the speed at which it moves, and the injuries to the whole body which results in little chance of survival (on railway lines, the chances are much lower than within the railway station—6% vs. 16%) and quick death [22,23,24]. Usually, accidents are noticed by train staff. Suicidal victims jump or fall directly in front of an oncoming train in the presence of passengers or other observers, lie face down across the track, and wait for the train to arrive or start, sit or kneel on the tracks, walk along the tracks, or on the tracks and are hit by a moving train [25]. Such incidents are a traumatic experience for the train driver, as well as for responders involved in removing human remains, and for possible witnesses of the incident. According to data from the World Health Organization, 18–21% of healthy people experience a severe reaction to stress (bewilderment, anger, despair) and even posttraumatic stress disorder (PTSD) after traumatic events. Additional psychological support for bystanders, responders, and train drivers is needed in Poland. It is estimated that only 30% of train drivers have received professional psychological help after their train struck and killed a suicide victim [26]. 

In railway accidents, an investigation is launched to clarify whether it was a railway suicide, an unfortunate accident (most often the result of crossing the tracks in an unauthorized place), or murder.

The classification of railway accidents in Poland is regulated by relevant legislation [27,28]. Suicides and suicide attempts constitute a separate group in the statistics and are not included in the classification of railway accidents. The change in classification of the direct cause of railway accidents to “railway suicide” is made only based on a decision of the Public Prosecutor’s Office.

Procedures for the investigation and proceedings for railway accidents caused by suicides and railway line crossings in prohibited locations vary from country to country. The minimum requirements for data collection are specified in the Community Railway Safety Directive. The Directive also requires the establishment of special investigating bodies. In Poland, it is the State Commission for Railway Accident Investigation which independently conducts its investigation of the circumstances of the incident [29]. The results of the investigation must be reported to the state authorities which deal with data collection. An important issue in the investigation is to establish the motive for choosing to die by conducting psychological interviews with people closely related to the victim, analysis of documents produced by the victim, e.g., a farewell letter, or examination of the circumstances immediately preceding the event.

In recent years, despite the preventive measures taken to reduce the number of suicides, there has been an increase in their number [30]. A report published in 2018 concluded that in the European Union, suicides represented 73% of the total number of people killed in rail transport. Cyprus, Latvia, Greece, Poland, Lithuania, Romania are countries where the percentage of suicides in railway accidents was less than 50%. In Norway, Ireland, Slovenia, and the Netherlands, suicides were predominant [31].

This study aims to determine important risk factors affecting railway suicide in Poland and their relation to the state of alcohol intoxication of the victims, and also the relationship between ethyl alcohol consumption and the phenomenon of suicide. 

## 2. Materials and Methods

### 2.1. Materials

The authors analyzed the documentation obtained from the Department of Forensic Medicine of the Medical University of Warsaw in the form of death registers and forensic medical records concerning examination and autopsy. A sum of 135 pedestrian victims of railway accidents from the Warsaw area were identified, including 60 victims of railway suicide. Finally, only suicide victims were included in the analysis. The analysis included gender and age of the victims, cause of death, date of autopsy (month and year), blood alcohol concentration (BAC), muscles, and vitreous bodies, all of which were confirmed by toxicological examination performed by gas chromatography. This information was supplemented with data from police notes attached to the protocols. The data obtained in this way were the basis for classifying the case to the group of suicide deaths or its rejection from this group. In cases of unexplained circumstances of death, i.e., when, based on the available information, it was not possible to determine whether the death was a result of a suicide act, accident, or homicide (the so-called “open verdict”), suicide was assumed as the cause of death, which is confirmed in practice through the judiciary courts [32,33]. Based on the results of the toxicological tests, suicide victims were divided into two groups, i.e., sober (BAC = 0.00‰) and under the influence of alcohol at the time of the incident (BAC > 0.00‰).

### 2.2. Methods

To answer the research questions, statistical analyses were conducted using IBM SPSS Statistics, version 25. For frequency analysis, a series of chi-square tests of independence, a series of t-Student tests for independent samples, Pearson r correlation analysis, and logistic regression were performed. The significance level was set at α = 0.05. Results whose significance was within the range of 0.05 > *p* > 0.1 were considered significant for statistical trends. 

## 3. Results

Suicide victims constituted more than 44% (*n* = 60) of the victims of railway accidents recorded in the studied period. Almost 77% (*n* = 46) of suicide victims were men. The age of the victims varied, ranging from 17 to 89 years, with the average age being 45.38 ± 20.51 years. Alcohol was found in 50.00% (*n* = 30) of the victims, with the average alcohol level in the victims being 0.97 ± 1.12‰. The detailed results are presented in Table 1.

### 3.1. Gender

The analysis performed with the chi-square independence test did not show a statistically significant relationship between gender and alcohol, χ2 = 0.37, *p* = 0.542, φ = 0.08 (Table 1).

### 3.2. Age

As a result of the analysis conducted with the chi-square test, a statistically significant correlation between age and the presence of alcohol was found. Sober suicide victims were statistically significantly older than those under the influence of alcohol, χ2 = 15.67, *p* = 0.028, φ = 0.51. (Table 1).

### 3.3. Season

Chi-square analysis showed a significant correlation between the season and the presence of alcohol, χ2 = 7.77, *p* = 0.051, φ = 0.36 (Table 1). Based on adjusted residuals, a higher than expected number of victims died by suicide in the autumn among sober victims (2.7), and a lower than expected number of suicide victims died by suicide in the autumn among victims under the influence of alcohol (−2.7). 

#### The Concentration of Ethyl Alcohol

Based on the analysis performed with Student’s t-test for independent samples, no statistically significant difference in alcoholic strength between males and females was found, t = −1.37, *p* = 0.181, d = 0.63 (Table 1).

The analysis showed a significant positive linear relationship at the statistical trend level between alcohol level and age, r = 0.34, *p* = 0.068. The older the suicide victims were, the higher the alcohol level was. The relationship was moderately strong.

### 3.4. Regression Analysis

A logistic regression analysis was also conducted to determine important predictors for dying by suicide under the influence of alcohol. The applied regression model (Nagelkerke R2) proved to be well suited to the data, χ2 = 15.32, *p* = 0.009. It explained 30% of the variance.

Based on multidimensional analysis, age and season were found to have a significant impact on the probability of dying by suicide under the influence of alcohol. This probability decreases with the age of the victims. It is also significantly lower in autumn (Table 2).

## 4. Discussion

Every year, there are many railway accidents caused by suicides and pedestrians crossing tracks in prohibited places. The RESTRAIL project, launched in 2011 based on the International Union of Railways (UIC in France) initiative, has made it possible to collect and analyze a range of data from 17 organizations across Europe, including the Polish Railway Institute. The project demonstrated the scale of suicides and illegal intrusions onto railways and their consequences. Thus, it provided the railway sector with results that make it possible to determine optimal preventive solutions and measures for limiting the impact of these events [34].

Every year, European Union (EU) member states report around 3000 railway suicides, which represents approximately 8% of all suicides in Europe. This ratio varies significantly between member states, with rates ranging from 2 to 14% [35]. 

In our study, victims of railway suicides accounted for nearly half of the victims of railway accidents recorded during the period studied.

It is estimated that between 34% and 56% of suicide victims abuse or are addicted to alcohol, and the risk of suicide deaths in this group is between 2% and 3.4%, which is six times higher than in people without this diagnosis [36,37,38]. According to RESTRAIL, nearly 50% of all railway suicide victims were under the influence of alcohol, medication, or drugs, with those intoxicated with alcohol being mostly young men [35]. In our study, 50% of suicide victims were under the influence of alcohol at the time of the incident. In this group, the average alcohol concentration was 0.97 ± 1.12 (range: 0.80–4.20‰), which corresponds to alcohol intoxication, which is considered a significant risk factor for suicidal behavior. However, no statistically significant difference in alcohol concentration between women and men was found, which means that the average alcohol concentration in men and women was comparable, though the high concentrations in women (1.55 ± 0.47‰) are a worrying trend. Alcohol concentration correlated with the age of the victims, i.e., the older the victims were, the higher the alcohol concentration. This correlation may suggest a cause and effect relationship between alcohol abuse and suicide.

One of the main risk factors of suicide is the gender of victims [39,40,41]. According to RESTRAIL, the majority of railway accident victims, including suicide victims, were male [35]. Although the presented analysis did not demonstrate a statistically significant relationship between gender and the presence of alcohol, men were four times more frequently under the influence of alcohol at death than women. This condition may be explained by the fact that men abuse alcohol more often than women to alleviate so-called “psychological pain” [35,36,37,38]. Moreover, they are less willing to seek mental health services, which may lead to impulsive and self-aggressive behaviors [42]. The predominance of men abusing alcohol is a phenomenon that is mainly observed in high-income countries. In low-middle-income countries, the proportion of men to women is much lower, but there are large differences between regions and between countries. On a global scale, only Asian countries, including India and China, are considered exceptions where, in general, women die by suicide as often as men [3,43].

Differences in gender proportions are also related to age. There are many reasons why female and male suicide rates differ. These include gender equality, differences in coping with stress and conflict, the availability and preference of different ways of dying by suicide, the availability and patterns of drinking alcohol, and differences in the rates of seeking help for mental disorders by women and men.

Taking into account the age of the victims, suicide rates in almost all regions of the world are the highest among people aged 70 and over—in both men and women. Globally, suicide constitutes the second most common cause of death in the 15–29 age group [1]. The results of the RESTRAIL project show that in railway accidents caused by suicides and railroad crossings in prohibited places, the age of victims was 20–59 years [35]. 

In the study, sober suicide victims were statistically significantly older than those under the influence of alcohol (50.43 ± 24.26 vs 40.33 ± 14.63 years, respectively). Age also proved to be an important predictor of suicide among victims under the influence of alcohol in multidimensional analysis. The results obtained through multidimensional analyses predict the probability of dying by suicide under the influence of alcohol decreases with the age of the victims. Perhaps this fact can be explained by greater determination in older people. According to Bomba, for young people, suicide is a call for help, while for older people, it is only a call for death [44].

Numerous studies have examined the seasonal variability phenomenon of suicides. The peak for suicides can be observed in the spring months, although some analyses indicate that a peak can also be observed in autumn [45,46]. In the period covered by the RESTRAIL project, the highest number of railway suicides was recorded in April and the lowest in December. Meanwhile, no visible trend was observed in Scandinavia, for example, and in Turkey, more suicides were recorded in the summer months [35]. 

The results of the presented analysis confirm the phenomenon of seasonal variability of suicides. Unidimensional analysis showed a significantly higher number of deaths by suicide among sober victims in autumn (September, October, November) compared to other seasons. On the other hand, multidimensional analysis suggests that the season of the year is an important predictor for dying by suicide under the influence of alcohol. The probability of dying by suicide under the influence of alcohol is significantly lower in autumn. Perhaps greater autumn “activity” among sober victims may be associated with neurobiological factors, including serotonergic disorders, manifested by autumnal depression. Undoubtedly, identification of other risk factors, which coexist with the phenomenon of seasonal variability of suicides, would add to the knowledge base to be used in the prevention of railway suicides.

In summary, according to the WHO, suicidal behavior is an essential health and social problem. According to the Health for All in the 21st Century policy adopted by the WHO in 2013, this problem should be solved as soon as possible, including the early identification of important risk factors associated with dying by suicide, limiting access to various methods of dying by suicide, identifying high-risk social groups, strengthening self-help groups, and education [1]. Because railway track suicides occur quite regularly, and the number of suicides is significant, it is important to look for effective methods to counteract this phenomenon. These include the application of appropriate technical measures, e.g., closed-type stations, fences making it difficult for unauthorized persons to enter the tracks; as well as nontechnical measures, i.e., development of various strategies which may influence the occurrence of suicides, national plans taking into account the necessity to reduce the number of suicides, poster campaigns, education of railway staff, and influencing the media, so that through extensive reporting they discourage victims from imitating suicide attempts [35]. Preventive measures taken by the Polish railways are consistent with the conclusions of the RESTRAIL project. The Railway Institute, which participated in five work packages of this project, participated in the development of a web-based tool for disseminating and exploiting project results, collecting and providing information such as best practices, links, and contacts. However, for effective suicide prevention, it is first of all necessary to develop a National Suicide Prevention Program and create appropriate conditions for social non-governmental voluntary organizations to take leadership roles. Currently, there is only a working group at the Ministry of Health that deals with suicide.

Railway suicides are a difficult problem to monitor, requiring extensive cooperation between the authorities involved in post-accident investigations. Only a harmonized and regular exchange of information about such incidents can help to prevent them effectively, although it seems equally important to systematize the methods of their collection because official statistics on suicides in Poland currently come from two databases, the National Police Information System (polish KGP) and the Central Statistical Office [47]. Unfortunately, railway suicides are described in these statistics as “throwing oneself under a moving vehicle”, which may be too much of a generalization, and do not specify the actual method of dying by suicide. Since September 2016, the Group for Registration of Suicidal Behavior at the Working Team for the Prevention of Depression and Suicides at the Public Health Council has been working on improving the reporting of suicides and suicide attempts in Poland. As a result of the work, KGP modified the National Register of Suicide Attempts and Suicides as of 1 January 2017, allowing for the comparison of certain data with the Central Statistical Office database. An obstacle to both databases is the lack of a Universal Electronic System for Registration of the Population (polish PESEL) in KSIP 10. The aim should be to centrally register suicidal behavior by creating a National Register of Suicide Attempts and Suicides, which will allow for the development of appropriate preventive measures, implementation, and evaluation [48,49,50].

## 5. Limitations

The study does not include all risk factors for suicide. Due to the existence of cases that, for various reasons, have not been reported as suicides, the results obtained should be treated as an attempt to describe the phenomenon, not as a representation of absolute numerical values. The main limitation of this study is its single institutional nature, since only a sum of 135 pedestrian victims of railway accidents from the Warsaw area were identified, including 60 victims of railway suicide. 

The authors’ experience gained from this study will form the basis for planned future studies. At the same time, it serves a wider standardization of the research tool used. Due to the essence of the problem, it seems necessary to conduct further, in-depth research in this area.

## 6. Conclusions

The observed relationship between age and the presence of alcohol in suicide victims may be the cause of railway suicides. Knowledge of the mechanisms of seasonal variability of suicidal behavior can help to develop effective strategies to prevent railway suicides. It is necessary to improve the methods of reporting suicides on railways, as only reliable statistics provide the possibility of assessing both the scale of the problem and the effectiveness of actions taken. 

## Figures and Tables

**Table 1:** Characteristics of railway suicide victims.

Variables	No Alcohol Group *n* = 30 (50.00%)	Alcohol Group *n* = 30 (50.00%)	Total *n* = 60 (100.00%)
Gender *n (%)*			
Women	8 (26.70)	6 (20.00)	14 (23.33)
Men	22 (73.30)	24 (80.00)	46 (76.67)
Age *n (%)*			
Minimum	17	18	17
Maximum	89	85	89
Mean age (M)	50.43 ± 24.26	40.33 ± 14.63	45.38 ± 20.51
Median (Me)	52	40	44
Season *n (%)*			
Spring	5 (16.70)	10 (33.30)	15 (25.00)
Summer	4 (13.30)	7 (23.30)	11 (18.34)
Autumn	15 (50.00)	5 (16.70)	20 (33.33)
Winter	6 (20.00)	8 (26.70)	14 (23.33)
Ethyl alcohol concentration *(‰)*			
Minimum	0	0.80	
Maximum	0	4.20	
Mean (M)	0	0.97 ± 1.12	
Women	0	1.55 ± 0.47	
Men	0	2.02 ± 0.81	

**Table 2:** Predictors in the logistic regression model predicting the probability of dying by suicide under the influence of alcohol.

Predictors	B *	*p*
Age	−0.043	0.017
Winter		0.025
Spring	0.620	0.457
Summer	0.778	0.404
Autumn	−1.677	0.039
Gender	0.180	0.803
Fixed	2.124	0.024

* Coefficient B (−) lower probability. Coefficient B (+) higher probability
